# Discrepancy of particle passage in 101 mask batches during the first year of the Covid-19 pandemic in Germany

**DOI:** 10.1038/s41598-021-03862-z

**Published:** 2021-12-29

**Authors:** Lukas T. Hirschwald, Stefan Herrmann, Daniel Felder, Anna M. Kalde, Felix Stockmeier, Denis Wypysek, Michael Alders, Maik Tepper, Jens Rubner, Peter Brand, Thomas Kraus, Matthias Wessling, John Linkhorst

**Affiliations:** 1 AVT.CVT - Chair of Chemical Process Engineering, RWTH Aachen University, Forckenbeckstraße. 51, 52074 Aachen, Germany; 2 DWI - Leibniz Institute for Interactive Materials, Forckenbeckstr. 50, 52074 Aachen, Germany; 3 Institute for Occupational, Social and Environmental Medicine, RWTH Aachen University Hospital, Pauwelstr. 30, 52074 Aachen, Germany

**Keywords:** Chemical engineering, Occupational health, Characterization and analytical techniques

## Abstract

During the first wave of Covid-19 infections in Germany in April 2020, clinics reported a shortage of filtering face masks with aerosol retention> 94% (FFP2 & 3, KN95, N95). Companies all over the world increased their production capacities, but quality control of once-certified materials and masks came up short. To help identify falsely labeled masks and ensure safe protection equipment, we tested 101 different batches of masks in 993 measurements with a self-made setup based on DIN standards. An aerosol generator provided a NaCl test aerosol which was applied to the mask. A laser aerosol spectrometer measured the aerosol concentration in a range from 90 to 500 nm to quantify the masks’ retention. Of 101 tested mask batches, only 31 batches kept what their label promised. Especially in the initial phase of the pandemic in Germany, we observed fluctuating mask qualities. Many batches show very high variability in aerosol retention. In addition, by measuring with a laser aerosol spectrometer, we were able to show that not all masks filter small and large particles equally well. In this study we demonstrate how important internal and independent quality controls are, especially in times of need and shortage of personal protection equipment.

## Introduction

Aerosols are heterogeneous mixtures of solid or liquid suspended particles in a gas. Liquid aerosols, produced via talking, coughing, sneezing, breathing, and singing^1–5^ can contain contaminants such as viruses^6^. Exposure to such aerosols can significantly increase the risk of infection. To prevent infection, personal protection equipment (PPE) can be used to shield aerosol emissions from others, as well as for self-protection^7–9^.

One kind of PPE is face masks, which have proven to be an effective measure against aerosol infection and are common protection equipment for medical personnel^3,10–12^. Two types of masks are used primarily: surgical masks and filtering masks (also known as respirators). As surgical masks are mainly designed for bacteria and droplet rejection, the retention of aerosols is low^13^. In contrast, filtering masks are designed as depth filters for the retention of aerosols, where an additional size-dependent adsorption of particles down to 0.1–0.3 $\upmu \text{m}$ through electrostatic interactions of the incorporated electret material^3,14–17^. The masks geometry is designed for a tighter fit around the face, reducing leakage compared to surgical masks. As filtering masks are originally designed to protect by minimizing the inhaled concentration of airborne particles in environments with high loads of fine particulates, various countries have classified them according to their filtration efficiency. European standards include the filtering facepiece class 3 (FFP3) with a maximum passage of 1% and the filtering facepiece class 2 (FFP2) with a maximum passage of 6% according to DIN EN 149^18^. With similar testing standards and product requirements to FFP2^18–22^, the US standard defines N-series (N95) with a maximal passage of 5% according to NIOSH-42CFR84^22^. The Chinese standard defines a KN-series (KN95) with a maximal passage of 5% according to GB 2626-2006^21^. In the usual certification processes, the respective mask is initially certified for a period of up to five years and requires intermediate quality control^23^.

Due to the fast global spreading of the new coronavirus (Sars-CoV-2) at the beginning of 2020^24^, the World Health Organization (WHO) declared COVID-19 a worldwide pandemic on March 11, 2020^25^. Although transmission of coronaviruses may occur by direct contact or contact with contaminated surfaces^3,26^, the most probable way of infection is via droplets and aerosols^2,3,26,27^. While the typical droplet size of infectious aerosols was found to be in the range of 1–20 $\upmu \text{m}$ and larger^2,4,5,28–32^, the size of the new coronavirus is approximately 120 $\text{nm}$^3,4,27,33^. Thus, due to the recommendation of health organizations, governments around the world stipulate using medical masks in public and, more importantly, for healthcare workers and infectious patients^11,34–36^.

These worldwide recommendations added up to a drastic increase in PPE demand and a sudden shortage of face masks^11,33,37^. Hence, production of all types of face masks, especially of higher protection classes such as FFP2, N95 and KN95, needed to be suddenly increased many times over. The enormous demand for face masks and hurried expansion of the production capacity led to varying product qualities for the end customer in several cases. Due to the supply shortage of filtering masks, new manufacturers entered the market, while at the same time, many types of masks were only insufficiently controlled due to the limited time. This allowed counterfeit, uncontrolled and incorrectly labeled products to enter the market so that buying new masks became dangerous^12,38–44^. As a result, researchers around the world started to work on options for the reusability of filtering face masks^45–48^.

Also, different studies on the measurement of particle retention were reported^4,12^. Lam et al.^12^ measured 160 brands of surgical masks and found that 48.8% of the tested masks did not fulfill their standard. Schilling et al.^4^ presented a low-cost setup for evaluating filtration performance and breathability of filtering face masks. The authors performed material tests of 47 non-regulated, commercially available masks and saw a poor performance for more than half of the tested masks. These studies show that companies, hospitals, and others require validation of medical mask filtration performance.

In this study, we present a method to evaluate the retention performance of masks and identify non-compliant masks. The custom measurement setup allowed us to quickly and non-destructively measure a large number of masks, especially during the early months of the pandemic. We tested 5–50 mask samples of 101 batches by various producers, totaling 993 measured masks. The high number of measurements subsequently allowed robust statistical analysis of the results. Filtering face masks were tested according to the European standard with additional insights in further product properties, such as overall rejection, size-dependent rejection, a production period dependent product quality, and a systematic investigation of potential erroneous parts of the mask (material, weld joints, fit).

We show that there are substantial differences in filtration performances in all investigated protection classes. Only about a third of all tested mask batches fulfilled their standard. Also, we found a tendency of lower protection performance for face masks that were produced at the beginning of the COVID-19 pandemic.

## Results and discussion

### Setup and validation

The three international standards used for PPE certification require a flame photometer for particle passage determination. This device monitors the overall passage as total mass summed up across all particle sizes, which is considered sufficient as a lethal dose reference. In contrast, a laser spectrometer gives information on the number of particles passing the sample as a function of particle size. By mass-weighted averaging of passing particles, redundant information to a flame photometer can be acquired. The auxiliary individual particle size data gained via the laser spectrometer is displayed in the supplementary information attached to this manuscript. Additionally, information on the dependency of passage on particle size allows for further statistical analysis of mask performance. The laser spectrometer is used to extract the efficiency in particle removal for single particle size classes, as the international standards vary in the monitored particle sizes. However, the European Union decided to regard the respective international standards as equivalent to the FFP2 standard^49^. This is why all masks tested in the scope of this study have been subject to a unified testing procedure. As the use of a laser spectrometer is a deviation from the designated standards, in this study, we, first, use a flame photometer and a laser spectrometer in redundancy for method validation and, second, investigate the size-dependent protection performance of different mask batches.

**Figure 1:**
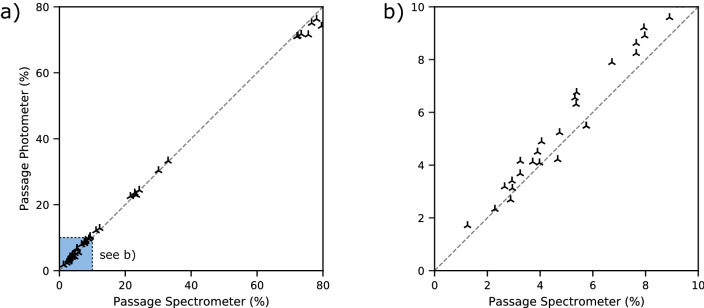
Passage detected with the flame photometer plotted over the passage detected with the laser spectrometer to demonstrate the validity of comparing different PPE measuring standards. (**a**) The entire range from 0 to 100% passage is plotted to show the overall agreement of both measuring methods. (**b**) Magnification of the range from 0 to 10% in which the retention limits of all standards are located.

To validate the standalone use of the laser spectrometer, a flame photometer was used in parallel to quantify the aerosol passage. The discrete measurements of the laser spectrometer were converted to an overall mass passage value to allow comparability and validation described in the “Methods” section. Figure 1 shows the overall passage of both detectors plotted against each other for validation. Ideally, both detectors measure the same passage and therefore all values would lie on the bisecting line.

The agreement between the two measurement methods is assessed with Lawrence Lin’s concordance correlation coefficient $\rho _c$^50,51^ (see Eq. 4). The coefficient equals 1 if all points lie on the bisecting line and decreases for increasing deviations from this line. For the full data range (Fig. 1a) from 0 to 100% a $\rho _c$ of 0.9987 is obtained. In the clinically relevant range for protective face masks of up to 10% passage (Fig. 1b), a value of 0.95 is observed. This indicates a good agreement between the measurements by flame photometry and laser spectroscopy. Therefore, we consider both methods equivalent.

### Filtration performance

**Figure 2:**
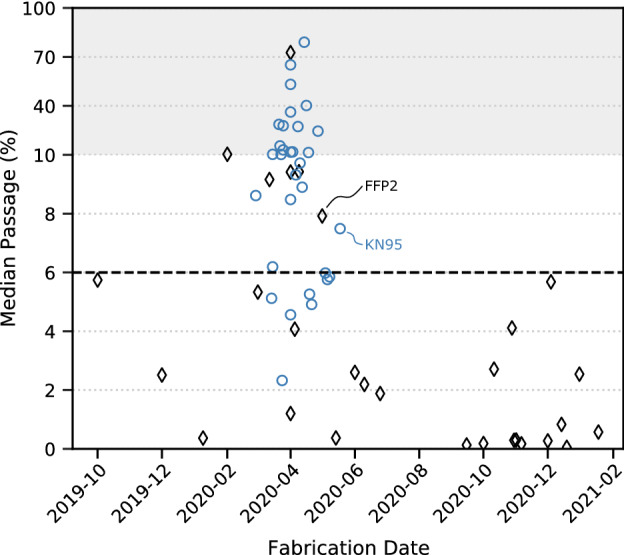
Mean aerosol passage over time for () FFP2 and () KN95 certified masks. The gray background indicates a change in scale in the y-axis. The dashed line marks the 6% specification according to DIN EN 149.

During the first month that the pandemic hit Germany, the setup was established, and performance evaluation of different face masks was started to help communal organizations provide safe equipment to their staff. Therefore, our results indicate changes in the overall mask quality over the course of the pandemic. After the slackening of the pandemic situation, we used the acquired base of data for statistical evaluation to help evaluate mask protection performance and enhance product quality tests in the future.

Figure 2 shows the mean passage as a measure of quality of all tested FFP2 and KN95 grade masks fabricated between October 2019 and February 2021, each data point representing one mask batch. While FFP2 grade masks had production dates spanning the entire measurement period, the tested KN95 grade masks were produced only between February 2020 and June 2020, resulting from a rapidly increased availability during that period.

The mask performance results are interpreted in close temporal relation with the course of the pandemic, increase in production capability, and mask availability. The strong decrease in mask quality starting February 2020 correlates with the significant increase in demand during the first Covid-19 wave and the following increase in production. Our results show that the increased production was accompanied by a decrease in mask quality. For KN95 masks, our results are limited to the production period from February 2020 to June 2020 and do not allow us to draw conclusions on quality beyond that period. The limited availability of FFP2 masks during that time led to an increase in the use of KN95 masks. KN95 masks were gradually superseded by FFP2 masks on the German market as soon as their availability stabilized.

Most tested FFP2 batches comply with the specification over the whole observation time. However, between February 2020 and June 2020, which coincides with the first wave of the pandemic in Germany, the required specification is not met by about 50% of the batches, with one batch being completely out of specification (> 70%). In contrast, most KN95 mask batches (92%) do not comply with the 6% passage specification. Hence, our results show a strong dependence of mask performance on the temporal market situation.

**Figure 3:**
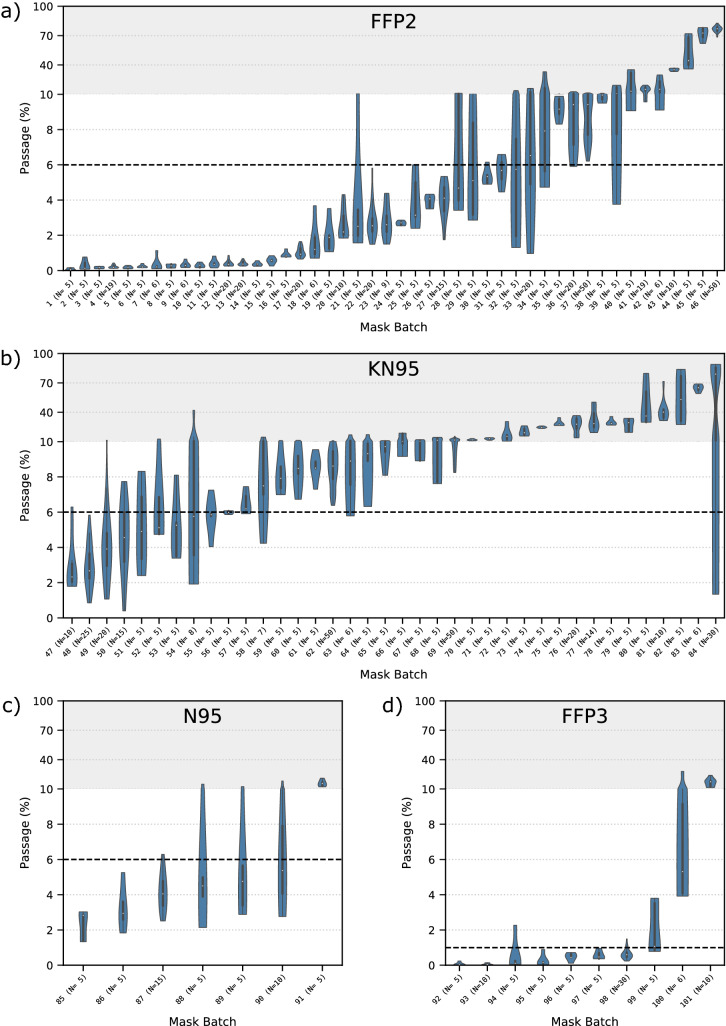
Violin plots for the 101 evaluated mask batches with 993 individual measurements. The dashed lines indicate the limit according to DIN EN 149. All measurements and the data evaluation were done according to DIN EN 149 and DIN EN 13274-7. The gray background indicates a change in scale in the y-axis. An extensive explanation of violin plots can be found in the “Materials and methods” section. : median,  : kernel density plot,  : interquartile range (boxplot from upper to lower quartiles), | minimum to maximum value excluding outliers.

Irrespective of the mask production period, the mask performance was measured for each batch individually. The individual mask batch results of particle passage are displayed in Fig. 3. The passage of particles is displayed as violin plots, which are similar to box plots, except that their thickness at different passages reflects the probability density of the data. In all violin plots, the passages are averaged over all particle size classes individually detected by the laser aerosol spectrometer, allowing for a conclusive evaluation of the spread between individual masks. This way, the data displayed here is consistent with the data assessment in standardized mask certification. The respective standardized maximal passage is indicated by a dashed line, while the axis is shrunk for passages above 10%.

In Fig. 3a, passages of all investigated mask batches officially certified as FFP2 are plotted. The violin plots allow for an evaluation of the spread of passage between individual masks in the measurement. Strong deviations in qualities can be observed between the different mask batches. In general, three main groups of masks can be identified. First, masks that show a passage of particles well below the standardized limit of 6% (batches 1–20). This excellent product performance allows for statistical deviations while still meeting the standard for every single mask. Second, a group of masks that shows particle passage in the range of the standardized limit can be identified (batches 21–33). Most products of this group are featured by high variability in passage between individual masks tested. This effect of high performance deviations between masks demonstrates a significant discontinuity in product quality among single masks. With less than 20 masks being tested in the standardized certification process, there is a high statistical risk of masks being certified that exhibit particle passages higher than the maximum allowed passage. Hence, the variability in product performance for some product types underlines the need for representative sample sizes to evaluate the product quality on a statistically reliable basis. Third, a group of masks can be identified that show particle passages way above the allowed limit of 6% (batches 34–46).

Figure 3b shows the violin plots of particle passage for the masks according to the KN95 standard. In contrast to the results shown in Fig. 3a, no masks with particle passages well below the standardized limit of 6% were found. In the group with the best particle retention performance, masks that show particle passage in the range of the standardized limit can be identified (batches 47–57), showing high deviations between individual masks. A second category of KN95 masks was found with particle passages in the order of 9% (batches 58–69). The other KN95 masks can be clustered in a third group that exhibits very high particle passages (batches 70–84). However, it must be noted that the KN95 products investigated in this study were all produced in the first period of the pandemic, in which also many products certified by other standards were not able to meet the standard’s requirements.

The particle passages of N95 and FFP3 mask batches are displayed in Fig. 3c, d, respectively. For N95 products, most investigated batches show particle passages widely spread around the standardized limit (batches 86–90). Only one batch of masks shows passages distinctly below the standardized limit of 6% (batch 85), and one batch stands out with a passage of more than 10% (batch 91). For FFP3 products, two of the tested batches exhibit very low particle passages (batches 92–93). Half of the as FFP3 certified mask batches show comparatively high spreads below or slightly over the standardized limit of 1% (batches 94–98). A third group of products, again, is featured by high particle passages of many times higher than the allowed maximum (batches 99–101).

The certification standards require the masks to pass a mass fraction criterion of the impinged aerosol, which is in accordance with the current understanding of toxicity referring to a certain dose^52^. The analysis of the total mass, in turn, reduces the influence of small aerosol particles on the measured performance result. Hence, masks only retaining large particles might still pass the criterion, while the passage of small particles might be well above the limit. This certification design is suitable for PPE applications in medical environments, where a maximum virus load must be prevented. In this context, the total amount of virus load scales with the aerosol particle volume. Hence, a mass-weighted protection performance evaluation is a good measure for effective human protection. In mask applications for environments containing high loads of fine particulates, however, particle passage sensitivity towards smaller particle sizes might cause severe differences in protection performance. In these cases, smaller particles tend to be of an elevated hazardous potential^53^. Hence, the retention of small particles in these use cases is of higher importance.

**Figure 4:**
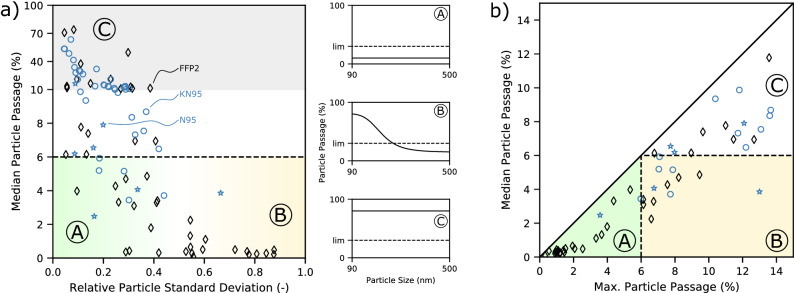
Particle size-dependent retention for () FFP2, () KN95, and () N95 masks. (**a**) Particle median passage over the relative standard deviation of passage over particle sizes. A, B, and C mark regimes for classification of the mask performance of fully norm compliant (A), partially compliant (B) and non-compliant (C) mask batches. The dashed line marks the 6% specification according to DIN EN 149. For the three regimes (A), (B), and (C), the three small graphs exemplarily show the particle passage over particle size. The dotted line represents the maximum allowed passage of 6%. (**b**) Particle median passage over highest passage of all particle size classes within one batch. The dashed lines mark the 6% specification according to DIN EN 149.

To investigate this crucial mask performance parameter, we used the particularity of the laser aerosol spectrometer of measuring particle passages as a function of the particles’ diameters. For statistical evaluation of the size-dependent spread in retention, we calculated the standard deviation between all measured passages of different particle size classes. We determined the particle classes’ passages by averaging the respective size class passages over all masks contained in one batch. In Fig. 4a, high median passages indicate low performance at most particle sizes. A small relative standard deviation of particle passage reflects a uniform particle retention behavior over particle sizes. In contrast, a high relative standard deviation of particle passage implies an increased spread in the passage for different particle sizes. In these cases, the measured particle size distributions indicate that the mask still retains large particles while smaller aerosols pass through.

Concerning particle passage deviation for different particle sizes, three categories can be identified. Most favorable are masks of category A, featured by a low median passage and low relative deviation of the particle passage across all investigated particle sizes. These masks uniformly retain particles and aerosols across the whole measured particle size range well below the standard’s limit. Masks of category B show a low median passage but high deviations as a function of particle size. These products tend to retain larger particles while being more permeable to smaller ones. Masks of category C are least favorable as they show a high median particle passage. The low values for the relative deviation of passage show that these mask batches tend to be permeable across the whole span of particle sizes investigated.

In Fig. 4b, the particle median passage for all samples of one product batch and over all particle size classes is plotted over the highest particle passage of all particle size classes within one batch (max. particle passage). This depiction allows for a distinction between masks of three types; (A) masks that comply with the maximum passage for all particle size classes (median and maximum passage below the standardized overall maximum passage); (B) masks that show higher particle passages for some particle classes, but fulfill the required product quality of a maximum overall passage; and (C) masks that do not meet the standard’s requirements.

## Conclusion and outlook

Face masks are an effective tool to protect humans from aerosols that potentially contain viruses or other harmful substances. Depending on the desired application, different standards according to country of origin and product quality are defined. With the rise of the Sars-CoV-2 pandemic, the demand for face masks drastically increased in early 2020. Most severe was a scarcity in face masks of higher protection classes such as FFP2, KN95, or N95.

The drastic increase in face mask demand led to a significant expansion of face mask production capacity. However, the standardized certification process does not require a renewed certification after such process capacity adaptions. As a result, incertitude evolved for end customers and raised the need for extensive studies on face mask protection behavior. In this study, we investigated 101 batches of face masks by various manufacturers. For protection performance evaluation, we used a setup complying with the defined standards for testing filtration performance during the face mask certification process. Our setup can be simplified by replacing cost-intensive equipment by low-cost alternatives and can thus be easily replicated with little financial resources. The aerosol generator could be replaced by ambient particles and the laser aerosol spectrometer could be exchanged for simple Rayleigh scattering detectors or a clean room particle counter.

Comparing the filtration performance of face masks on average over the production period, a tendency of lower protection performance can be identified for FFP2, KN95, N95, and FFP3 face masks produced in the early phase of the pandemic. However, all face masks investigated with a production date in the second half of 2020 or beginning of 2021 (FFP2, N95, FFP3) meet the standards according to their certification. Overall, tremendous differences in mask performances were observed in all protection classes. Only about a third of all mask batches investigated met the standard requirements, while another third showed particle passages slightly over the standardized maximum passage. In turn, this means that a third of all mask batches investigated in this study exhibited a particle passage significantly higher than the required standard, indicating a severe lack in protection efficiency.

Further, using a laser spectrometer in our setup allowed for an additional evaluation of the particle passage as a function of applied particle size. This analysis revealed additional differences in product quality among FFP2, KN95 and N95 mask batches. While some mask types retain all sizes of particles equally well, other mask types show a decreased particle retention for small particles. While this complies with face mask applications for medical environments, it might be undesirable for applications in environments with high loads of fine particulates.

## Materials and methods

### Experimental setup

**Figure 5:**
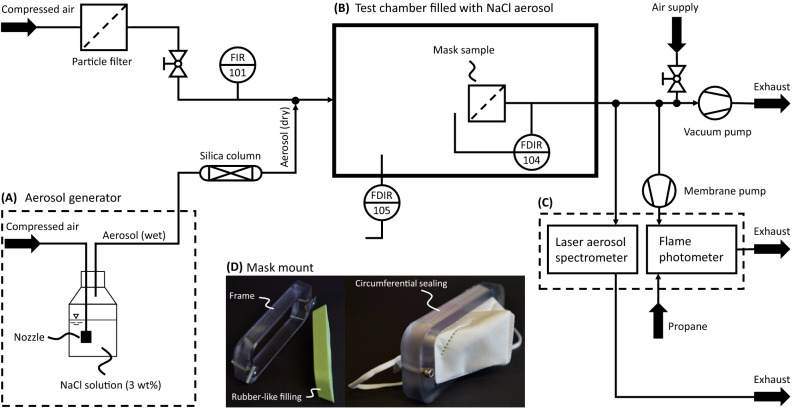
Flowchart of mask testing rig. (**A**) Aerosol generation. (**B**) Test chamber, pressure drop across the mask and pressure in the test chamber relative to ambient pressure are measured. (**C**) Aerosol detection with a laser aerosol spectrometer as well as a flame photometer. (**D**) Mask mount consisting of a stable frame and a rubber-like filling. Mask installed into the mount shown on the right side.

In this work, an experimental setup based on DIN EN 149 and DIN EN 13274-7 is used to test all presented face masks. The setup consists of three main parts: an aerosol generator (A), a test chamber (B), and an aerosol analyzer (C), as shown in Fig. 5.

To test face masks concerning their aerosol passage, a defined aerosol is necessary to get comparable results consistently. A test following DIN EN 149^18^ requires two different test aerosols, a sodium chloride aerosol and a paraffin oil aerosol. The sample needs to be exposed to the aerosols at an airflow rate of 95 L min$^{-1}$. To test a high amount of masks with a simple test and with the actual use of the mask known, in this work, only sodium chloride aerosols were used to determine the aerosol passage of face masks. The precise protocol for the aerosol passage test with sodium chloride and, thus, the requirements for the aerosol generator are stated in DIN EN 13274-7^54^. To obtain the required aerosol with a median in particle size distribution between 0.06 and 0.10 $\upmu \text{m}$, an aerosol concentration between 4 and 12 mg m$^{-3}$ in the test chamber, and a relative humidity of 40% or less at 22 C a ’Portable Test Aerosol Generator 3073’ (TSI Inc., Shoreview (US)) is used. The aerosol generator produces the aerosol by pumping compressed air through a nozzle submerged in a 3 wt% sodium chloride solution. To dry the wet aerosol produced by the generator, the stream is passed through a silica column. In this work, a ‘3062-NC Diffusion Dryer’ (TSI Inc., Shoreview (US)) is used. The 12 $\upmu \text{m}$ tubing between the aerosol generator and the silica column is heated to prevent condensation of wet aerosol on the tubing walls, especially during the long experiments needed to test large numbers of masks in a row. A ‘T-570’ (Winkler AG, Heidelberg (DE)) temperature controller with an ‘HBSI 100 W’ (Horst GmbH, Lorsch (DE)) heating tape is used. Condensation would result in a decreased aerosol concentration and possibly a change in particle size distribution leading to insignificant test results. The dried aerosol stream is mixed with additional compressed air, as shown in the left part of Fig. 5 to reach 95 L min$^{-1}$. The additional compressed air is filtered through a submicro filter ‘SMF 0002’ (Landefeld GmbH, Kassel (DE)) prior to the mixing point to prevent changes in the test aerosol caused by particles in the compressed air.

The generated aerosol is fed into a test chamber (see Fig. 5B). The test chamber needs to be closed against the environment and must provide an appropriate and sealed fixation for face masks. The airflow needs to be withdrawn from the test chamber behind the face mask, and the withdrawn air needs to be fed to suitable analyzers. In this work, a ‘Smart Store 45L’ (Orthex Group, Lohja (FI)) storage box is used as a test chamber. The storage box comes with a lid and is stable and sealed when a vacuum of up to 20 mbar under ambient pressure is applied. All tubing is connected to the test chamber with suitable IQS fittings. To perfuse the tested face mask with the required flow rate of 95 L min$^{-1}$ a vacuum pump behind the sealed face mask withdraws the air from the test chamber. A ‘WD 5 P’ (Kärcher SE & Co. KG, Winnenden (DE)) is used in this work. The complete air needs to pass through the mask to leave the test chamber. The tests are conducted with a pressure difference between − 3 and − 7 mbar compared to ambient pressure. The pressure difference between the inside of the test chamber and ambient pressure is monitored by a differential pressure sensor (105). A second differential pressure sensor (104) measures the pressure drop of the face mask.

The test chamber needs to be divided into two parts by the face mask: the part in front of the mask, where the test aerosol is fed into, and a part behind the mask, where the air is withdrawn from the test chamber and fed to the aerosol analyzer to measure the aerosol concentration. To avoid leakage of particles, the mask needs to be tightly sealed. A mask mount was specifically designed to ensure tight sealing of the mask and for easy operation during sample change (see Fig. 5D).

The mask mount consists of a stable, leak-proof 3D-printed frame (Polyjet Objet Eden 260V 3D printer by Stratasys, Minneapolis, US) and a silicone inlay. The inlay was produced by using the frame as a mold. The face mask is clamped between frame and inlay for tight sealing during the measurement. The design of the mask holder ensures an identical filter area for all masks in one batch. To account for different face mask shapes, slight deviations (aspect ratio and circumference) from the original design were fabricated to ensure identical filtration area and tight sealing. Thus, the surface area of each mask during the particle passage test was proportional to its surface area. Every mask mount was once validated against a measurement of a circular, punched-out filter sample in an o-ring sealed stainless steel filter holder. During a measurement, the pressure difference across the mask was measured. If the pressure difference was close to zero, the fit of the mask in the holder was checked and the measurement was repeated. We consider this measurement method as an improvement compared to the standard procedure of the German regulatory authority, as a fixed, circular surface area for the test of the filtration performance of each mask, independent of the surface area of the mask is used.

To analyze the aerosol concentration, DIN EN 13274-7 states an aerosol flame photometer with sample ports in front of the face mask and behind the face mask. In this work, a ‘FP 8400’ (Krüss Optronic GmbH, Hamburg (DE)) is used (see Fig. 5C). The flame photometer measures the concentration of sodium chloride in a gas stream by combustion in a flame. The flame is created by the combustion of propane with filtered and compressed air. Photo diodes detect the color change of the flame due to the combustion of sodium. The aerosol-containing sample flow is withdrawn from the tubing behind the test chamber by a membrane pump (KNF Neuberger GmbH, Freiburg (DE)), as shown in the right part of Fig. 5, and fed to the flame photometer. Furthermore, a laser aerosol spectrometer (LAS) ‘3340 A’ (TSI Inc., Shoreview (US)) is used for aerosol concentration measurements (see Fig. 5C). The LAS counts particles flowing through a measurement volume by measuring light scattering induced by particles crossing a laser beam. Thus, the LAS measures the number of particles and the related particle size distribution in the sample flow. The used LAS measures 99 particle size classes of 4.5% class width between 90 nm and 7.5 $\upmu \text{m}$. The LAS was connected to the outlet tubing of the test chamber, next to the sample port for the flame photometer. The LAS is equipped with a sample pump. Thus, no additional pump is needed.

### Experimental method

In the experimental setup, two measurements are necessary to evaluate the filtration performance of a face mask. The first measurement is a reference measurement, obtaining information on the aerosol in the test chamber. Therefore the mask mount is placed in the test chamber without a face mask installed. This measurement is also used to validate the generated aerosol and particle concentration in the test chamber with DIN EN 149. During the measurement, the test chamber is closed. The aerosol concentration and particle counts over one minute are measured for five consecutive minutes using the flame photometer and LAS. The first two minutes of each measurement are excluded from the evaluation of the mask’s filtration performance. As flow rates are slow compared to the length of tubing, this time is needed to reach equilibrium in the whole system.

After the reference measurement, the experiment with a mounted mask is performed. The sample holder is placed in the test chamber after checking for tight sealing of the mask. The filtration performance of the face mask is then calculated by using the collected data of minutes three to five. For evaluation of the filtration performance, the test measurement is compared to the reference measurement. When using the LAS, a correction term is used to calculate mass concentrations of the aerosol. This calculation procedure is described in the next section.

### Data evaluation

The detected mass of particles $m_i$ per particle class *i* is calculated from the LAS data by averaging the particle count $N_{i}$ per size over three minutes multiplied by the individual particle mass normalized by the mean sample volume flow ${\dot{V}}_{\text{sample}}$:

1 $$\begin{aligned} m_i = \frac{N_{i}}{{3}\,min} \cdot \frac{d_{\text{min},i}^3 \cdot \pi }{6} \cdot \rho \cdot \frac{1}{{\dot{V}}_\text{sample}} \end{aligned}$$

Here, the particle mass is calculated as the particle’s minimum volume with diameter $d_{\text{min}}$. The density $\rho$ cancels out when calculating the particle passage per size:

2 $$\begin{aligned} P_i = \frac{m_{\text{filter},i}}{m_{\text{reference},i}} \end{aligned}$$

The variables $m_{\text{filter},i}$ and $m_{\text {reference},i}$ are the particle mass per size with and without an installed filter, respectively. For the comparison to the results from the flame photometer, the mean particle passe is calculated as:

3 $$\begin{aligned} P_\text {total} = \frac{\sum _i m_{\text {filter},i}}{\sum _i m_{\text {reference},i}} \end{aligned}$$

The concordance correlation coefficient^51^ between two measurement methods *x* and *y* is calculated as

4 $$\begin{aligned} \rho _c&= \frac{2s_{xy}}{s_x^2 + s_y^2 + ({\bar{x}}-{\bar{y}})} \end{aligned}$$

where $s_x$ and $s_y$ are the variances of the data set *x* and *y* and $s_{xy}$ is the covariance. ${\bar{x}}$ and ${\bar{y}}$ are the arithmetic means.

Statistical evaluation was done in Python using pandas^55,56^ and seaborn^57^, visualizing the results in Matplotlib^58^. Violin plots were created with the standard seaborn implementation. The white dot in the center of each plot is the median of the measurement series. Surrounding the white dot is a small boxplot chart with a black rectangle showing the end of the upper and lower quartiles. The adjacent black line extends from the minimum to the maximum excluding any outliers. Around the boxplot diagram the blue the kernel density plot is shown, which represents the distribution of the measurements. The wider the kernel density plot, the more measurements are in this region, with each data point equally weighted. The number of measurements per batch is shown on the X-axis in parentheses.

## Supplementary Information

Supplementary Information.

Supplementary Data.
