# Supplementary material for: Discrepancy of particle passage in 101 mask batches during the first year of the Covid-19 pandemic in Germany
Source: Sci Rep. 2021 Dec 29;11:24490. doi: 10.1038/s41598-021-03862-z (PMC8716525; doi:10.1038/s41598-021-03862-z)
Supplement: Supplementary file 2 — Supplementary Data. [file 41598_2021_3862_MOESM2_ESM.zip › SI/Overview.pdf]

Batch Number	Standard	Number of Samples (N)	Median Passage (%)	Mask Manufacturer	Mask Name	Fabrication Batch	Fabrication Date
**1**	FFP2	5	0,05	Henan Aklly Filter Engineering Co., Ltd. - China	An ke lin - KZ888E	2012321	2020-12-19
**2**	FFP2	5	0,13	FarStar Medical GmbH - China	Series 30000 - 34100	3820BW	2020-09-15
**3**	FFP2	5	0,17	Anshun Health and Medical Technology Co., Ltd. - China	AKF6002	0120201103	2020-11-06
**4**	FFP2	19	0,18	Handan Hengyong Protective & Clean Products Co., Ltd. - China	HY9320	20285	2020-10-01
**5**	FFP2	5	0,18	Shenzhen HJR Electronics Technology Co., Ltd. - China	HJR-CN99-11		
**6**	FFP2	5	0,2	Guangdong Golden Leaves Technology Development Co., Ltd. - China	8865		
**7**	FFP2	6	0,2	Zhejiang Baiyi Medical Technology Co., Ltd. - China	Lemoat BY01		
**8**	FFP2	5	0,27	Handan Hengyong Protective & Clean Products Co., Ltd. - China	HY9320	20345	2020-12-01
**9**	FFP2	6	0,29	Kunshan Jinfujie Precision Mould Co., Ltd. - China	JFJ-302	201030001	2020-10-30
**10**	FFP2	5	0,3	Handan Hengyong Protective & Clean Products Co., Ltd. - China	HY9322	20315	2020-11-01
**11**	FFP2	5	0,35	Guangdong Kingfa Sci. & Tech. Co., Ltd. - China	KF-A F10(SC)		
**12**	FFP2	20	0,36	FIT Farm Innovation Team GmbH - Germany	FIT F246	2A05B04C09Q06J05E05	2020-01-09
**13**	FFP2	20	0,36	FIT Farm Innovation Team GmbH - Germany	FIT F246		
**14**	FFP2	5	0,37	3M	Aura 9322+		2020-05-14
**15**	FFP2	5	0,57	Shandong Daddy's Choice Health Science and Technology Co., Ltd - China	DADDY'S CHOICE Purism Protective Face Mask	01138644	2021-01-18
**16**	FFP2	5	0,83	Hangzhou Filtech Intelligent Co., Ltd. - China	FILTECH F861	205101	2020-12-14
**17**	FFP2	20	0,92	Hunan Dreaming Cloud E-Commerce Co., Ltd. - China	HYGISUN HS0501A		
**18**	FFP2	6	1,2	Zender Germany - Germany	CPA001 NR		2020-04-01
**19**	FFP2	5	1,88	Guangzhou Carrot Mall Network Technologies Co., Ltd. - China	IRYS-01	HLB20200625	2020-06-25
**20**	FFP2	10	2,19	SPRO Medical Products (Xiamen) Co., Ltd. - China	GL001A (no valve)	2005090	2020-06-10
**21**	FFP2	5	2,51	Uvex - Germany	uvex silv-Air 2200 - cup style		2019-12-01
**22**	FFP2	20	2,54	Dongguan Missadola Technology Co., Ltd. - China	Miramask 2626-2	202312	2020-12-31
**23**	FFP2	9	2,6	SPRO Medical Products (Xiamen) Co., Ltd. - China	GL001A (no valve)	2005084	2020-06-01
**24**	FFP2	5	2,71	Gaomi Morning Glory Footwear Co., Ltd. - China	M2004	#20201015	2020-10-11
**25**	FFP2	5	3,14	Shanghai Zhongzhi Health Articles Co., Ltd. - China	ZH3310		
**26**	FFP2	5	4,07	Shanghai Zhongzhi Health Articles Co., Ltd. - China	ZH3310	20200403	2020-04-05
**27**	FFP2	15	4,11	Hangzhou Filtech Intelligent Co., Ltd. - China	FILTECH F860	204403	2020-10-28
**28**	FFP2	5	4,68		Bursch 80610	20185031	
**29**	FFP2	5	5,1	Berner Trading Holding GmbH - Germany			
**30**	FFP2	5	5,33	Technaxx Deutschland GmbH & Co. KG - Germany	LifenaXX LX-016		2020-03-01
**31**	FFP2	5	5,68	Univent Medical GmbH - Germany	ATEMIOUS PRO - Art. 2001		2020-12-04
**32**	FFP2	5	5,74	ASATEX - Germany	FMP2	D1000670	2019-10-01
**33**	FFP2	20	6,52				
**34**	FFP2	5	7,92	Suzhou Teyin Nonwoven Co., Ltd. - China	TY0929V	974279-2000456-17	2020-05-01
**35**	FFP2	5	9,16		Dust Mask 3 - DM019	20200312	2020-03-12
**36**	FFP2	20	9,41	Gaomi Chenheng Labor Protection Products Co., Ltd. - China	KN95 Face Mask		2020-04-01
**37**	FFP2	50	9,43	Shandong C.I.R.S Garments Co., Ltd. - China	Leishide LSD007		2020-04-09
**38**	FFP2	5	9,96	National High-Tech Enterprise Chengde Technology Co., Ltd. - China	Zhong Jian Le - Folding Protective Mask		
**39**	FFP2	5	10,34	Suzhou Sanical Protective Product Manufacturing Co., Ltd. - China	Benehal 6112	600317	2020-02-01
**40**	FFP2	5	13,16	KOLIBRI GmbH - Germany	MASK		
**41**	FFP2	19	14,99	SPRO Medical Products (Xiamen) Co., Ltd. - China	GL001A (no valve)		

Batch Number	Standard	Number of Samples (N)	Median Passage (%)	Mask Manufacturer	Mask Name	Fabrication Batch	Fabrication Date
**42**	FFP2	6	15,22	Handan Hengyong Protective & Clean Products Co., Ltd. - China	HY8620		
**43**	FFP2	10	35,11	Zhejiang Zhuji Industrial Park - China	3DKN95 protectivemask		
**44**	FFP2	5	44,37	VIP Mask - Tureky	Solunum Maskesi		
**45**	FFP2	5	72,58		104BA	04/26/2020	2020-04-01
**46**	FFP2	50	77	BSI Group The Netherlands B.V. - Netherlands	SAFE		
**47**	KN95	10	2,33	Garry Galaxy Biotechnology Co., Ltd. - China	TrueTone Technology Respirator Mask, Size: Adult	201202	2020-03-24
**48**	KN95	25	2,67	3M China Co., Ltd. - China	9501V+		
**49**	KN95	20	3,91				
**50**	KN95	15	4,56	Jinhua Jinyi Welding Protective Tools Co., Ltd. - China	JY-5232A	202004	2020-04-01
**51**	KN95	5	4,91	Tengfei Technology Co., Ltd. - China	HB TF-003	2503202011	2020-04-21
**52**	KN95	5	5,12	Lanshan Shendun Technology Co., Ltd. - China	Lamdown SD-KN95		2020-03-14
**53**	KN95	5	5,26	Tengfei Technology Co., Ltd. - China	Effective Particle Filtering Mask - Earloop	2503202011	2020-04-19
**54**	KN95	8	5,76	Zhejiang RunKang Medical Equipment Co., Ltd. - China	BRK002 - 3D daily protective mask (non-medical)	20200506B	2020-05-06
**55**	KN95	5	5,85	Chengdu Baiyu Medical Supplies Co., Ltd. - China	Particle filtering half mask	20200508	2020-05-08
**56**	KN95	5	5,98	Ningbo Chengmei Medical Products Co., Ltd. - China	Hainmed	CMPJ0401	2020-05-04
**57**	KN95	5	6,19	Tongcheng Aimei Labor Protection Products Co., Ltd. - China	Henghao KN95 Face Masks	2020/03/15	2020-03-15
**58**	KN95	7	7,49	Jiangxi Guoyou Medical Technology Co., Ltd. -	GY-01, C-shaped mask	2020051801	2020-05-18
**59**	KN95	5	7,92	Foshan Lechen Hygienic Products Co., Ltd. - China	KU001		
**60**	KN95	5	8,48			04/26/2020	2020-04-01
**61**	KN95	5	8,5		PM2.5		
**62**	KN95	50	8,62				2020-02-28
**63**	KN95	6	8,9	Shandong Huishoutang Pharmaceutical Co., Ltd. - China	KN95 Respirator	20200405	2020-04-12
**64**	KN95	5	9,32	Lanshan Shendun Technology Co., Ltd. - China	Lamdown SD-KN95	2020-04-06	2020-04-06
**65**	KN95	5	9,72	Guangzhou Mingyu Medizintechnik Co., Ltd. - China	Hanging Ear		2020-04-10
**66**	KN95	5	10,23	Lanshan Shendun Technology Co., Ltd. - China	Lamdown SD-KN95		2020-03-23
**67**	KN95	5	10,3	Tongcheng Aimei Labor Protection Products Co., Ltd. - China	Henghao KN95 Face Masks	2020/03/15	2020-03-15
**68**	KN95	5	11,42	Yiwu Biweikang Labor Protection Products Co., Ltd. - China	Bi Wie Kang - KN95 Face Mask - 9600 Filter Type	YM20200416	2020-04-18
**69**	KN95	50	11,68	Jiangsu Nanfang Medical Co., Ltd. - China	Non-medical KN95, Earloop Style	20200408	2020-04-01
**70**	KN95	5	11,8	Henan Yubei Sanitary Materials Co., Ltd. - China		48200402	2020-04-03
**71**	KN95	5	13,03	Guangdong HuiSen New Material Technology Co., Ltd. - China	KN95 Particulate Respirator		2020-03-25
**72**	KN95	5	15,47	Shenzhen Yun Tongda Technology & Service Co., Ltd. - China	FM80	2020-03-22	2020-03-22
**73**	KN95	5	19,1		Sius		
**74**	KN95	5	24,53	Tong Cheng Xin Sheng Kang Labor Protection Products Co., Ltd. - China		20200427	2020-04-27
**75**	KN95	5	27,33	Zhejiang Wharney Daily Chemical Co., Ltd. - China	Disposable protective mask (non-medical)	20200408	2020-04-08
**76**	KN95	20	27,83	CTT Co., Ltd - China	Dust Mask	29002	2020-03-25
**77**	KN95	14	28,52	Shenzhen Anhuacheng Safety Technology Co., Ltd. - China	KN95 Stereo Respirator		
**78**	KN95	5	28,65	Shenzhen Fittop Health Technology Co., Ltd. - China	P-Mask FM80		2020-03-21
**79**	KN95	5	29,38		KN95 Mask with valve		
**80**	KN95	5	36,22		HUIJUN		2020-04-01
**81**	KN95	10	40,21				2020-04-16
**82**	KN95	5	53,16	Xinpu Safety Products (Shenzhen) Co., Ltd. - China	KN95 Protective Mask		2020-04-01

Batch Number	Standard	Number of Samples (N)	Median Passage (%)	Mask Manufacturer	Mask Name	Fabrication Batch	Fabrication Date
**83**	KN95	6	65,1		104BA	26.4.20	2020-04-01
**84**	KN95	30	78,98	Shandong Huishoutang Pharmaceutical Co., Ltd. - China	KN95 Respirator	20200406	2020-04-14
**85**	N95	5	2,83	Shanghai Dasheng Health Products Manufacture Co., Ltd. - China	DTC3W	200402	2020-04-01
**86**	N95	5	2,94	Shanghai Dasheng Health Products Manufacture Co., Ltd. - China	DTC3B	200402	2020-04-01
**87**	N95	15	4,05	Makrite Industries Inc. - Taiwan	SEKURA-321, TC-84A-6660		
**88**	N95	5	4,5		SOUND	BFD0324N	2020-03-01
**89**	N95	5	4,76	Suzhou Sanical Protective Product Manufacturing Co., Ltd. - China	Benehal MS6115L	600615	2020-02-01
**90**	N95	10	5,38	Makrite Industries Inc. - Taiwan	9500-N95	060620	2020-06-06
**91**	N95	5	15,59	Makrite Industries Inc. - Taiwan	TC-84A-5411	040220	
**92**	FFP3	5	0,02	Handan Hengyong Protective & Clean Products Co., Ltd. - China	HY9330	20225	2020-08-05
**93**	FFP3	10	0,04	Fido Masks Co., Ltd. - Taiwan	F333V	BEL2001470	2020-05-01
**94**	FFP3	5	0,11	Makrite Industries Inc. - Taiwan	FIT-P3DSL		2020-06-01
**95**	FFP3	5	0,16	Handan Hengyong Protective & Clean Products Co., Ltd. - China	HY9332	20315	2020-11-01
**96**	FFP3	5	0,44	ASATEX - Germany	FMP3V	D1000546	2019-08-01
**97**	FFP3	5	0,47	Kunshan Jinfujie Precision Mould Co., Ltd. - China	JFJ-1603V		
**98**	FFP3	30	0,6	Foshan Nanhai Plus Medical Co., Ltd. - China	ESMOO CP-N95F (FFP3)	30057062	2020-03-17
**99**	FFP3	5	1,07	MFA - Solunum Koruyucu Maskeler - Turkey	Prestige Series P-367		
**100**	FFP3	6	5,31	Zender Germany - Germany	CPA3		2020-07-01
**101**	FFP3	10	17,38		CNEKTP-3		
