# Supplementary material for: Discrepancy of particle passage in 101 mask batches during the first year of the Covid-19 pandemic in Germany
Source: Sci Rep. 2021 Dec 29;11:24490. doi: 10.1038/s41598-021-03862-z (PMC8716525; doi:10.1038/s41598-021-03862-z)
Supplement: Supplementary file 2 — Supplementary Data. [file 41598_2021_3862_MOESM2_ESM.zip › SI/Supplementary-Information.pdf]

Supplementary Information

101 Filtering Face Mask Batches – A Comprehensive Study on Particle Passage

Lukas T. Hirschwald[1,♥], **Stefan Herrmann**[1,♥], **Daniel Felder**[1,2,♥], **Anna M. Kalde**[1,2,♥], **Felix Stockmeier**[1,2,♥], **Denis Wypysek**[1,2,♥], **Michael Alders**[1], **Maik Tepper**[1,2], **Jens Rubner**[1], **Peter Brand**[3], **Thomas Kraus**[3], **Matthias Wessling**[1,2], **and John Linkhorst**[1,*]

[1]RWTH Aachen University, AVT.CVT - Chair of Chemical Process Engineering, Forckenbeckstraße. 51, 52074 Aachen, Germany
[2]DWI - Leibniz Institute for Interactive Materials, Forckenbeckstr. 50, 52074 Aachen, Germany
[3]RWTH Aachen University Hospital, Institute for Occupational, Social and Environmental Medicine, Pauwelstr. 30, 52074 Aachen, Germany
[♥]these authors contributed equally to this work
[*]manuscripts.cvt@avt.rwth-aachen.de

Dataset

The dataset, including all raw data and supplementary information, is available at https://doi.org/10.18154/RWTH-2021-05390.

Data Overview

This section explains all the general information about the masks and is intended to give the reader an overview of the data. On the following pages, the information is presented in tabular form. For better reusability, the data is also available as Excel (.xlsx), comma-separated values (.csv), and JavaScript Object Notation (.json). The overview data can be found under the name `Overview.*`. Since packaging was not available for each mask, not all information could be fully captured. Only explicit information that could be found on the masks or respective packages was included. The individual columns of the data as a table contain the following information:

Column	Data type	Description
Batch Number	integer	Consecutive numbering of all measured mask batches (1-101).
Standard	string	The standard according to which the respective batch was certified (FFP2, FFP3, KN95, N95).
Number of Samples (N)	integer	The number of individual masks (samples) measured in each batch.
Median Passage (%)	float	The median passage in percent of the respective batch (0-100%).
Mask Manufacturer	string	The manufacturer printed on the mask or mask packaging. Where possible, the manufacturer was given instead of the distributor.
Mask Name	string	The name/model/label of a mask.
Fabrication Batch	string	The batch number indicated on the mask/packaging by the manufacturer.
Fabrication Date	ISO 8601 date	The date of manufacture indicated on the mask/package by the manufacturer (YYYY-MM-DD).

In the comma-separated values (.csv) file, all values of the above-described columns are separated by commas. Therefore the decimal point is used for floating-point numbers.

The JavaScript Object Notation has the following data structure:

```
{
    "1":{
        "Standard":string,
        "Number of Samples (N)":integer,
        "Median Passage":float,
        "Mask Manufacturer":string,
        "Mask Name":string,
        "Fabrication Batch":string,
        "Fabrication Date":ISO 8610 date
    },
    "2":{
        ...
    },
    ...
    "101":{
        ...
    }
}
```

Batch Number	Standard	Number of Samples (N)	Median Passage (%)	Mask Manufacturer	Mask Name	Fabrication Batch	Fabrication Date
1	FFP2	5	0,05	Henan Aklly Filter Engineering Co., Ltd. - China	An ke lin - KZ888E	2012321	2020-12-19
2	FFP2	5	0,13	FarStar Medical GmbH - China	Series 30000 - 34100	3820BW	2020-09-15
3	FFP2	5	0,17	Anshun Health and Medical Technology Co., Ltd. - China	AKF6002	0120201103	2020-11-06
4	FFP2	19	0,18	Handan Hengyong Protective & Clean Products Co., Ltd. - China	HY9320	20285	2020-10-01
5	FFP2	5	0,18	Shenzhen HJR Electronics Technology Co., Ltd. - China	HJR-CN99-11		
6	FFP2	5	0,2	Guangdong Golden Leaves Technology Development Co., Ltd. - China	8865		
7	FFP2	6	0,2	Zhejiang Baiyi Medical Technology Co., Ltd. - China	Lemoat BY01		
8	FFP2	5	0,27	Handan Hengyong Protective & Clean Products Co., Ltd. - China	HY9320	20345	2020-12-01
9	FFP2	6	0,29	Kunshan Jinfujie Precision Mould Co., Ltd. - China	JFJ-302	201030001	2020-10-30
10	FFP2	5	0,3	Handan Hengyong Protective & Clean Products Co., Ltd. - China	HY9322	20315	2020-11-01
11	FFP2	5	0,35	Guangdong Kingfa Sci. & Tech. Co., Ltd. - China	KF-A F10(SC)		
12	FFP2	20	0,36	FIT Farm Innovation Team GmbH - Germany	FIT F246	2A05B04C09Q06J05E05	2020-01-09
13	FFP2	20	0,36	FIT Farm Innovation Team GmbH - Germany	FIT F246		
14	FFP2	5	0,37	3M	Aura 9322+		2020-05-14
15	FFP2	5	0,57	Shandong Daddy's Choice Health Science and Technology Co., Ltd - China	DADDY'S CHOICE Purism Protective Face Mask	01138644	2021-01-18
16	FFP2	5	0,83	Hangzhou Filtech Intelligent Co., Ltd. - China	FILTECH F861	205101	2020-12-14
17	FFP2	20	0,92	Hunan Dreaming Cloud E-Commerce Co., Ltd. - China	HYGISUN HS0501A		
18	FFP2	6	1,2	Zender Germany - Germany	CPA001 NR		2020-04-01
19	FFP2	5	1,88	Guangzhou Carrot Mall Network Technologies Co., Ltd. - China	IRYS-01	HLB20200625	2020-06-25
20	FFP2	10	2,19	SPRO Medical Products (Xiamen) Co., Ltd. - China	GL001A (no valve)	2005090	2020-06-10
21	FFP2	5	2,51	Uvex - Germany	uvex silv-Air 2200 - cup style		2019-12-01
22	FFP2	20	2,54	Dongguan Missadola Technology Co., Ltd. - China	Miramask 2626-2	202312	2020-12-31
23	FFP2	9	2,6	SPRO Medical Products (Xiamen) Co., Ltd. - China	GL001A (no valve)	2005084	2020-06-01
24	FFP2	5	2,71	Gaomi Morning Glory Footwear Co., Ltd. - China	M2004	#20201015	2020-10-11
25	FFP2	5	3,14	Shanghai Zhongzhi Health Articles Co., Ltd. - China	ZH3310		
26	FFP2	5	4,07	Shanghai Zhongzhi Health Articles Co., Ltd. - China	ZH3310	20200403	2020-04-05
27	FFP2	15	4,11	Hangzhou Filtech Intelligent Co., Ltd. - China	FILTECH F860	204403	2020-10-28
28	FFP2	5	4,68		Bursch 80610	20185031	
29	FFP2	5	5,1	Berner Trading Holding GmbH - Germany			
30	FFP2	5	5,33	Technaxx Deutschland GmbH & Co. KG - Germany	LifenaXX LX-016		2020-03-01
31	FFP2	5	5,68	Univent Medical GmbH - Germany	ATEMIOUS PRO - Art. 2001		2020-12-04
32	FFP2	5	5,74	ASATEX - Germany	FMP2	D1000670	2019-10-01
33	FFP2	20	6,52				
34	FFP2	5	7,92	Suzhou Teyin Nonwoven Co., Ltd. - China	TY0929V	974279-2000456-17	2020-05-01
35	FFP2	5	9,16		Dust Mask 3 - DM019	20200312	2020-03-12
36	FFP2	20	9,41	Gaomi Chenheng Labor Protection Products Co., Ltd. - China	KN95 Face Mask		2020-04-01
37	FFP2	50	9,43	Shandong C.I.R.S Garments Co., Ltd. - China	Leishide LSD007		2020-04-09
38	FFP2	5	9,96	National High-Tech Enterprise Chengde Technology Co., Ltd. - China	Zhong Jian Le - Folding Protective Mask		
39	FFP2	5	10,34	Suzhou Sanical Protective Product Manufacturing Co., Ltd. - China	Benehal 6112	600317	2020-02-01
40	FFP2	5	13,16	KOLIBRI GmbH - Germany	MASK		
41	FFP2	19	14,99	SPRO Medical Products (Xiamen) Co., Ltd. - China	GL001A (no valve)		

Batch Number	Standard	Number of Samples (N)	Median Passage (%)	Mask Manufacturer	Mask Name	Fabrication Batch	Fabrication Date
**42**	FFP2	6	15,22	Handan Hengyong Protective & Clean Products Co., Ltd. - China	HY8620		
**43**	FFP2	10	35,11	Zhejiang Zhuji Industrial Park - China	3DKN95 protectivemask		
**44**	FFP2	5	44,37	VIP Mask - Tureky	Solunum Maskesi		
**45**	FFP2	5	72,58		104BA	04/26/2020	2020-04-01
**46**	FFP2	50	77	BSI Group The Netherlands B.V. - Netherlands	SAFE		
**47**	KN95	10	2,33	Garry Galaxy Biotechnology Co., Ltd. - China	TrueTone Technology Respirator Mask, Size: Adult	201202	2020-03-24
**48**	KN95	25	2,67	3M China Co., Ltd. - China	9501V+		
**49**	KN95	20	3,91				
**50**	KN95	15	4,56	Jinhua Jinyi Welding Protective Tools Co., Ltd. - China	JY-5232A	202004	2020-04-01
**51**	KN95	5	4,91	Tengfei Technology Co., Ltd. - China	HB TF-003	2503202011	2020-04-21
**52**	KN95	5	5,12	Lanshan Shendun Technology Co., Ltd. - China	Lamdown SD-KN95		2020-03-14
**53**	KN95	5	5,26	Tengfei Technology Co., Ltd. - China	Effective Particle Filtering Mask - Earloop	2503202011	2020-04-19
**54**	KN95	8	5,76	Zhejiang RunKang Medical Equipment Co., Ltd. - China	BRK002 - 3D daily protective mask (non-medical)	20200506B	2020-05-06
**55**	KN95	5	5,85	Chengdu Baiyu Medical Supplies Co., Ltd. - China	Particle filtering half mask	20200508	2020-05-08
**56**	KN95	5	5,98	Ningbo Chengmei Medical Products Co., Ltd. - China	Hainmed	CMPJ0401	2020-05-04
**57**	KN95	5	6,19	Tongcheng Aimei Labor Protection Products Co., Ltd. - China	Henghao KN95 Face Masks	2020/03/15	2020-03-15
**58**	KN95	7	7,49	Jiangxi Guoyou Medical Technology Co., Ltd. -	GY-01, C-shaped mask	2020051801	2020-05-18
**59**	KN95	5	7,92	Foshan Lechen Hygienic Products Co., Ltd. - China	KU001		
**60**	KN95	5	8,48			04/26/2020	2020-04-01
**61**	KN95	5	8,5		PM2.5		
**62**	KN95	50	8,62				2020-02-28
**63**	KN95	6	8,9	Shandong Huishoutang Pharmaceutical Co., Ltd. - China	KN95 Respirator	20200405	2020-04-12
**64**	KN95	5	9,32	Lanshan Shendun Technology Co., Ltd. - China	Lamdown SD-KN95	2020-04-06	2020-04-06
**65**	KN95	5	9,72	Guangzhou Mingyu Medizintechnik Co., Ltd. - China	Hanging Ear		2020-04-10
**66**	KN95	5	10,23	Lanshan Shendun Technology Co., Ltd. - China	Lamdown SD-KN95		2020-03-23
**67**	KN95	5	10,3	Tongcheng Aimei Labor Protection Products Co., Ltd. - China	Henghao KN95 Face Masks	2020/03/15	2020-03-15
**68**	KN95	5	11,42	Yiwu Biweikang Labor Protection Products Co., Ltd. - China	Bi Wie Kang - KN95 Face Mask - 9600 Filter Type	YM20200416	2020-04-18
**69**	KN95	50	11,68	Jiangsu Nanfang Medical Co., Ltd. - China	Non-medical KN95, Earloop Style	20200408	2020-04-01
**70**	KN95	5	11,8	Henan Yubei Sanitary Materials Co., Ltd. - China		48200402	2020-04-03
**71**	KN95	5	13,03	Guangdong HuiSen New Material Technology Co., Ltd. - China	KN95 Particulate Respirator		2020-03-25
**72**	KN95	5	15,47	Shenzhen Yun Tongda Technology & Service Co., Ltd. - China	FM80	2020-03-22	2020-03-22
**73**	KN95	5	19,1		Sius		
**74**	KN95	5	24,53	Tong Cheng Xin Sheng Kang Labor Protection Products Co., Ltd. - China		20200427	2020-04-27
**75**	KN95	5	27,33	Zhejiang Wharney Daily Chemical Co., Ltd. - China	Disposable protective mask (non-medical)	20200408	2020-04-08
**76**	KN95	20	27,83	CTT Co., Ltd - China	Dust Mask	29002	2020-03-25
**77**	KN95	14	28,52	Shenzhen Anhuacheng Safety Technology Co., Ltd. - China	KN95 Stereo Respirator		
**78**	KN95	5	28,65	Shenzhen Fittop Health Technology Co., Ltd. - China	P-Mask FM80		2020-03-21
**79**	KN95	5	29,38		KN95 Mask with valve		
**80**	KN95	5	36,22		HUIJUN		2020-04-01
**81**	KN95	10	40,21				2020-04-16
**82**	KN95	5	53,16	Xinpu Safety Products (Shenzhen) Co., Ltd. - China	KN95 Protective Mask		2020-04-01

Batch Number	Standard	Number of Samples (N)	Median Passage (%)	Mask Manufacturer	Mask Name	Fabrication Batch	Fabrication Date
**83**	KN95	6	65,1		104BA	26.4.20	2020-04-01
**84**	KN95	30	78,98	Shandong Huishoutang Pharmaceutical Co., Ltd. - China	KN95 Respirator	20200406	2020-04-14
**85**	N95	5	2,83	Shanghai Dasheng Health Products Manufacture Co., Ltd. - China	DTC3W	200402	2020-04-01
**86**	N95	5	2,94	Shanghai Dasheng Health Products Manufacture Co., Ltd. - China	DTC3B	200402	2020-04-01
**87**	N95	15	4,05	Makrite Industries Inc. - Taiwan	SEKURA-321, TC-84A-6660		
**88**	N95	5	4,5		SOUND	BFD0324N	2020-03-01
**89**	N95	5	4,76	Suzhou Sanical Protective Product Manufacturing Co., Ltd. - China	Benehal MS6115L	600615	2020-02-01
**90**	N95	10	5,38	Makrite Industries Inc. - Taiwan	9500-N95	060620	2020-06-06
**91**	N95	5	15,59	Makrite Industries Inc. - Taiwan	TC-84A-5411	040220	
**92**	FFP3	5	0,02	Handan Hengyong Protective & Clean Products Co., Ltd. - China	HY9330	20225	2020-08-05
**93**	FFP3	10	0,04	Fido Masks Co., Ltd. - Taiwan	F333V	BEL2001470	2020-05-01
**94**	FFP3	5	0,11	Makrite Industries Inc. - Taiwan	FIT-P3DSL		2020-06-01
**95**	FFP3	5	0,16	Handan Hengyong Protective & Clean Products Co., Ltd. - China	HY9332	20315	2020-11-01
**96**	FFP3	5	0,44	ASATEX - Germany	FMP3V	D1000546	2019-08-01
**97**	FFP3	5	0,47	Kunshan Jinfujie Precision Mould Co., Ltd. - China	JFJ-1603V		
**98**	FFP3	30	0,6	Foshan Nanhai Plus Medical Co., Ltd. - China	ESMOO CP-N95F (FFP3)	30057062	2020-03-17
**99**	FFP3	5	1,07	MFA - Solunum Koruyucu Maskeler - Turkey	Prestige Series P-367		
**100**	FFP3	6	5,31	Zender Germany - Germany	CPA3		2020-07-01
**101**	FFP3	10	17,38		CNEKTP-3		

Measurement Data

This section explains the data measured during our work. The entire data of 101 batches including all 993 measurements is available as Excel (.xlsx), comma-separated values (.csv), and JavaScript Object Notation (.json). The respective files are named `Data.*`. The individual columns of the data as a table contain the following information:

Column	Data type	Description
Batch Number	integer	Consecutive numbering of all measured mask batches (1-101).
Sample Name	string	The identifier of each sample as an aggregate of the batch number and consecutive numbering of all samples within the batch. The first three digits represent the batch number; the separator is an underscore (_); the last two digits represent the sample number within the batch (e.g. 001_01 to 001_05 for the 1st batch).
Mass-Weighted Mean Passage	float	The mass-weighted mean passage in percent of the respective sample (0-100%).
90 - 491.5	float	90 to 491.5 are the lower limits of the particles size classes in nanometer. The cells contain the passage in percent within the respective particle size class (0-100%).

In addition to the data in a single file, the measurements of the batches are also provided in individual files. The individual files are named after the respective batch `001.*` to `101.*`. Again, the data is available in the three formats .csv, .xlsx, and .json.

Comparison of Laser Spectrometer with Flame Photometer

In this section, the measurement data to validate the laser spectrometer against the flame photometer is presented. The data is available as Excel (.xlsx), comma-separated values (.csv), and JavaScript Object Notation (.json). The respective files are named `Validation.*`. As the flame photometer already outputs a single passage value per measurement for validation only the mass-weighted mean passage of the laser spectrometer is used. The individual columns of the data as a table contain the following information:

Column	Data type	Description
Batch Number	integer	Consecutive numbering of all measured mask batches (1-101).
Standard	string	The standard according to which the respective batch was certified (FFP2, FFP3, KN95, N95).
Passage Laser Spectrometer	float	The mass-weighted mean passage in percent of the respective sample measured with the laser spectrometer (0-100%).
Passage Flame Photometer	float	The passage in percent of the respective sample measured with the flame photometer (0-100%).

The JavaScript Object Notation file (`Validation.json`) is numbered consecutively from 0 to 43. These numbers do not correlate with the batch numbers, as the batch number is included in each data point separately. As flame photometer data was not recorded for each of the 101 batches, a different numbering was chosen. The file has the following data structure:

```
{
    "0":{
        "Batch Number":integer,
        "Standard":string,
        "Passage Laser Spectrometer":float,
        "Passage Flame Photometer":float
    },
    "1":{
        ...
    },
    ...
    "43":{
        ...
    }
}
```
